# Defining Culture Conditions for the Hidden Nitrite-Oxidizing Bacterium *Nitrolancea*

**DOI:** 10.3389/fmicb.2020.01522

**Published:** 2020-07-10

**Authors:** Eva Spieck, Katharina Sass, Sabine Keuter, Sophia Hirschmann, Michael Spohn, Daniela Indenbirken, Linnea F. M. Kop, Sebastian Lücker, Alejandra Giaveno

**Affiliations:** ^1^Department of Microbiology and Biotechnology, Universität Hamburg, Hamburg, Germany; ^2^Technology Platform Next Generation Sequencing, Heinrich Pette Institut, Hamburg, Germany; ^3^Department of Microbiology, IWWR, Radboud University, Nijmegen, Netherlands; ^4^PROBIEN (CONICET-UNCo), Departamento de Química, Facultad de Ingeniería, Universidad Nacional del Comahue, Neuquén, Argentina

**Keywords:** nitrification, nitrite oxidation, *Nitrolancea*, cultivation, wastewater treatment plant, geothermal springs

## Abstract

Nitrification is a key process for N-removal in engineered and natural environments, but recent findings of novel nitrifying microorganisms with surprising features revealed that our knowledge of this functional guild is still incomplete. Especially nitrite oxidation – the second step of nitrification – is catalyzed by a phylogenetically diverse bacterial group, and only recently bacteria of the phylum *Chloroflexi* have been identified as thermophilic nitrite-oxidizing bacteria (NOB). Among these, *Nitrolancea hollandica* was isolated from a laboratory-scale nitrifying bioreactor operated at 35°C with a high load of ammonium bicarbonate. However, its distribution remains cryptic as very few closely related environmental 16S rRNA gene sequences have been retrieved so far. In this study, we demonstrate how such thermophilic NOB can be enriched using modified mineral media inoculated with samples from a wastewater side-stream reactor operated at 39.5°C. Distinct cultivation conditions resulted in quick and reproducible high enrichment of two different strains of *Nitrolancea*, closely related to *Nl. hollandica*. The same cultivation approach was applied to a complex nitrite-oxidizing pre-enrichment at 42°C inoculated with biomass from a geothermal spring in the Copahue volcano area in Neuquen, Argentina. Here, an additional distinct representative of the genus *Nitrolancea* was obtained. This novel species had 16S rRNA and nitrite oxidoreductase alpha subunit (*nxrA*) gene sequence identities to *Nl. hollandica* of 98.5% and 97.2%, respectively. A genomic average nucleotide identity between the Argentinian strain and *Nl. hollandica* of 91.9% indicates that it indeed represents a distinct species. All *Nitrolancea* cultures formed lancet-shaped cells identical to *Nl. hollandica* and revealed similar physiological features, including the capability to grow at high nitrite concentrations. Growth was optimal at temperatures of 35–37°C and was strongly enhanced by ammonium supplementation. Genomic comparisons revealed that the four *Nitrolancea* strains share 2399 out of 3387 orthologous gene clusters and encode similar key functions. Our results define general growth conditions that enable the selective enrichment of *Nitrolancea* from artificial and natural environments. In most natural habitats these NOB apparently are of low abundance and their proliferation depends on the balanced presence of nitrite and ammonium, with an optimal incubation temperature of 37°C.

## Introduction

As important part of the global nitrogen cycle, nitrification converts reduced nitrogen species into oxidized forms, namely ammonium (−III) to nitrite (+III) and further to nitrate (+V). In wastewater treatment plants, nitrification is essential to remove N-compounds like ammonium and nitrite, and to provide nitrate as electron acceptor for denitrification. In agricultural, but also natural systems, nitrification causes nitrogen loss due to leaching of negatively charged nitrate ions and emission of gaseous N-compounds produced by denitrifying bacteria.

Both steps of nitrification are performed by distinct groups of bacteria or archaea (Koops and Pommerening-Röser, 2005; Spieck and Bock, 2005; Stieglmeier et al., 2014), but the traditional differentiation into ammonia and nitrite oxidizers has been overturned with the discovery of comammox *Nitrospira*, which can perform the complete oxidation of ammonia to nitrate (Daims et al., 2015; van Kessel et al., 2015).

Nitrite-oxidizing bacteria (NOB) from six genera belonging to four different phyla have been isolated so far. These are *Nitrobacter, Nitrotoga* and *Nitrococcus* within the *Alpha*-, *Beta*- and *Gammaproteobacteria*, respectively (Spieck and Bock, 2005; Alawi et al., 2007), *Nitrospira* and *Nitrospina*, which belong to separate phyla (Ehrich et al., 1995; Lücker et al., 2013), and the moderate thermophilic NOB *Nitrolancea*, which is phylogenetically affiliated with the *Chloroflexi* (Sorokin et al., 2012, 2014). The latter phylum also contains novel thermophilic nitrite oxidizers that have been highly enriched from hot springs at Yellowstone National Park (Spieck et al., 2020). Indications for further marine NOB have been obtained by metagenomics studies (Lüke et al., 2016; Ngugi et al., 2016; Sun et al., 2019).

*Nitrolancea hollandica* has been isolated from a lab-scale nitrifying bioreactor receiving a high load of ammonium bicarbonate and operating at 35°C (Vejmelkova et al., 2012). It has a high tolerance against nitrite as typical for *r*-strategists among the NOB (Andrews and Harris, 1986; Nowka et al., 2015), which appears to correlate with the cytoplasmic localization of the key enzyme nitrite oxidoreductase (NXR) (Spieck et al., 1998; Lücker et al., 2010). The genomic organization of the *nxr* genes in *Nitrolancea* is very similar to those in *Nitrobacter, Nitrococcus* (Sorokin et al., 2012) and the recently described *Candidatus* (*Ca.*) Nitrocaldera (Spieck et al., 2020). In contrast, NOB containing a periplasmic NXR like *Nitrospira* (Spieck et al., 1996) are mostly inhibited by high substrate concentrations (Off et al., 2010). This distinct *Nitrospira*-type NXR is closely related to that of anaerobic ammonium-oxidizing (anammox) bacteria (Lücker et al., 2010), while the cytoplasmic *Nitrobacter*-type NXR is closely related to dissimilatory nitrate reductases (NAR) of nitrate-reducing microorganisms (Kirstein and Bock, 1993).

The low substrate affinity of *Nl. hollandica* (K_m (__nitrite__)_ = 1 mM; Sorokin et al., 2012) posed the question of its natural niche. Nitrite is an intermediate of nitrification, denitrification and nitrate reduction to ammonium. It can accumulate when these processes are imbalanced, an effect that can be exaggerated by abiotic factors like pH, temperature or certain operational modes in reactor systems (Philips et al., 2002).

Temperature is a main factor shaping the community structure of nitrite-oxidizing bacteria and variations of this incubation parameter have resulted in the discovery of novel NOB (Alawi et al., 2007; Lebedeva et al., 2008). Nitrite oxidation at elevated temperature was found to be performed by *Nitrospira* (Lebedeva et al., 2005, 2011; Courtens et al., 2016), a common NOB in geothermal habitats (Marks et al., 2012; Edwards et al., 2013), or novel NOB of the *Chloroflexi* phylum (Spieck et al., 2020).

Based on the observed properties, the natural habitat of *Nl. hollandica* has been proposed to feature high temperatures as well as high ammonium and nitrite concentrations, as present for instance in compost or dung (Sorokin et al., 2012). *Nitrolancea*-like 16S rRNA gene sequences have only rarely been detected in specialized incubations of water or soil samples (Sorokin et al., 2018). These include soil columns used for aquifer treatment (Friedman et al., 2018), 1,3-dichloropropene fumigated soil (Fang et al., 2019), and manure composting (Zhang et al., 2016). A closely related 16S rRNA gene sequence (JN087902) has been detected in a SHARON (single reactor system for high activity ammonium removal over nitrite) reactor in Korea running at elevated temperature, a system with conditions comparable to the source of *Nl. hollandica*. Furthermore, recent surveys revealed the presence of this NOB in four Danish enhanced biological phosphorus removal (EBPR) systems and an N-removal plant in China (Speirs et al., 2019). Additionally, *Nitrolancea-*like 16S rRNA gene sequences have been found in reactors used for partial nitrification (Yu et al., 2018, 2020) and in free nitrous acid (FNA) treated wastewater (Wang et al., 2020). However, so far it is unclear what the lower temperature limit for proliferation of this moderate thermophilic NOB is and if *Nitrolancea* is present and active in full-scale nitrifying sludge systems especially as activated sludge does not contain ammonium or nitrite in the high concentrations apparently required by *Nitrolancea* to be competitive.

Apart from engineered systems, 16S rRNA gene sequences distantly related to *Nitrolancea* were found in warm natural environments, like the Atacama Desert (Orlando et al., 2010, EU603380) and the Kalahari (Elliott et al., 2014). These findings raise the question whether geothermal habitats might be suitable habitats for *Nitrolancea*, too. Therefore, in this study the geothermal area Las Máquinas in Argentina was investigated for the presence of nitrifying microorganisms. This region belongs to the Copahue-Caviahue system, which is mostly located in the Neuquen Province in Northern Patagonia and is characterized by a high biodiversity (Chiacchiarini et al., 2010; Urbieta et al., 2012, 2015a,b; Giaveno et al., 2013; Willis Poratti et al., 2016).

In the present study, three new representatives of *Nitrolancea* were selectively enriched under comparable conditions, and two pure cultures were obtained. Two of the enrichment cultures originated from a full-scale centrate treatment reactor at a wastewater treatment plant (WWTP) in Hamburg, Germany, and one from the geothermal Las Máquinas area in Argentina. The obtained species were compared to the type strain *Nl. hollandica* with regard to their genomic, phenotypic and physiological features.

## Materials and Methods

### Sampling

#### WWTP Hamburg-Dradenau, Germany

On May 22, 2017, a centrate treatment side-stream reactor (3300 m^3^) at the full-scale WWTP Hamburg-Dradenau was sampled (sample Z2; Supplementary Figure S1A). The centrate originated from centrifuged digested sludge and contained high ammonium (38 mM) and nitrite (44 mM), and low nitrate (9 mM) concentrations, and had a temperature of 39.5°C and a pH of 6.5–6.9 on the sampling day. In the treatment reactor, this liquid was mixed with activated sludge from the nitrification stage (approx. 30% v/v) and was supplied with 2 mg O_2_ l^–1^. The HRT was 1 day. The solids content and chemical oxygen demand (COD) amounted to 550 mg l^–1^ and 1750 mg l^–1^, respectively. Subsequent to this “Store and treat” (SAT) procedure, patent-registered by the HSE (Hamburger Stadtentwässerung), the reactor effluent was applied for anammox treatment.

#### Las Máquinas, Argentina

The Copahue volcano (37°51′S, 71°10.2′W; 2977 m a.s.l), the Rio Agrio (pH 0.5–7) and Caviahue Lake (pH 2.1–3.7) are geographical landmarks that frame a geothermal field, which is composed of five hydrothermal sites: Las Máquinas, Las Maquinitas, Anfiteatro, Termas de Copahue (in Argentina) and Chancho-Co (in Chile). The Las Máquinas area contains many thermal fluid emission systems, consisting of fumaroles, boiling-bubbling water and mud pools with wide ranges of pH (2–7) and temperature (30–90°C). The chemical composition of the fumaroles, which are mainly fed by meteoric water (Chiodini et al., 2015), showed that the main emitted gas is CO_2_, followed by minor concentrations of N_2_, H_2_, CH_4_, and H_2_S. CO and He concentrations are <1 ppm. Ammonium as a substrate for nitrification was detected in concentrations between 0 and 20 mM at different sampling sites of Las Máquinas, whereas nitrite and nitrate occurred between 0 and 6 μM (unpublished results).

In December 2008 (sample E2) and in March 2011 (sample A4), two mud pools in the geothermal area Las Máquinas, Argentina, were sampled. Both sampling sites were characterized by high sulfate concentrations (Supplementary Table S1). For sample E2 (Supplementary Figure S1B), red to brownish sediment was removed at a site that had a temperature of 40°C, a pH of 2.5–3.0 and an ammonium content of 0.6 mM. The sampling site of A4 (Supplementary Figure S1C) had a temperature of 45°C, a pH of 6.0 and ammonium was below the detection limit. Sample aliquots were transported at ambient temperature by courier to the University of Hamburg, Germany. Temperature, pH, and electric conductivity were measured *in situ* in each sample point using specific electrodes (Thermo Scientific Orion 5-Star Plus benchtop multifunction meter, electrode Orion 9142BN, Waltham, MA, United States). Water samples for metal analysis were filtered using 0.2 μm membrane filters (Cellulose acetate, Sartorius, Göttingen, Germany) and their concentrations (Supplementary Table S1) determined by atomic absorption spectrophotometry (AAS; Merodio and Martínez, 1985). To quantify anions, a standard turbidimetric assay for the determination of sulfate with BaCl_2_ (Kolmert et al., 2000) and a titration of chloride ions with mercuric nitrate solution using diphenyl carbazide indicator were used (APHA et al., 1992).

### Cultivation Media

The enrichments were performed in 0.05 to 0.15 l batch cultures in mineral salts medium with the following composition: 0.007 g l^–1^ CaCO_3_, 0.5 g l^–1^ NaCl, 0.05 g l^–1^ MgSO_4_ × 7 H_2_O, and 0.15 g l^–1^ KH_2_PO_4_ dissolved in distilled water. If not stated otherwise, 0.02–0.2 g l^–1^ NaNO_2_ (0.29 – 2.9 mM) were supplied as energy source. The trace element solution (1 ml l^–1^) contained 33.8 mg l^–1^ MnSO_4_ × H_2_O, 49.4 mg l^–1^ H_3_BO_3_, 43.1 mg l^–1^ ZnSO_4_ × 7 H_2_O, 37.1 mg l^–1^ (NH_4_)_6_Mo_7_O_24_, 973.0 mg l^–1^ FeSO_4_ × 7 H_2_O, and 25.0 mg l^–1^ CuSO_4_ × 5 H_2_O. The pH was adjusted to 8.0 and changed to 7.4–7.6 2 days after autoclaving. In order to enrich *Nitrolancea*, NH_4_Cl was added in concentrations between 0.5 and 10 mM (Supplementary Table S2). When bicarbonate (0.5 mM) was included, the pH had to be adjusted to 6.7 to reach a final pH of 7.4–7.6 after autoclaving. For growth of the centrate-derived cultures, and the Argentinian cultures from 2018 on, trace elements according to Widdel and Bak (1991) were added to the medium (1 ml l^–1^): 100 mg l^–1^ MnCl_2_ × 4 H_2_O, 30 mg l^–1^ H_3_BO_3_, 144 mg l^–1^ ZnSO_4_ × 7 H_2_O, 36 mg l^–1^ Na_2_MoO_4_ × 2 H_2_O, 2.1 g l^–1^ FeSO_4_ × 7 H_2_O, 2 mg l^–1^ CuCl_2_ × 2 H_2_O, 190 mg l^–1^ CoCl_2_ × 6 H_2_O, and 24 mg l^–1^ NiCl_2_ × 6 H_2_O.

### Enrichment

#### WWTP Hamburg-Dradenau

Two batches of mineral NOB medium (see above) containing 3 mM NaNO_2_ and 3 mM NH_4_Cl were inoculated (1% v/v) with the centrate sample Z2 on June 22, 2017, and incubated without agitation at 42°C and 46°C, respectively. A transfer was performed on October 23, 2017, and the incubation temperature was lowered to 37°C. In the following, the cultures grew very well and nitrite concentrations supplied to the medium were continuously increased (Supplementary Table S2).

#### Copahue – Argentina

A 0.5 ml aliquot of sample E2 (spring water with sediment) was inoculated into a 100 ml Erlenmeyer flask with 50 ml mineral unbuffered AOB medium (Krümmel and Harms, 1982) containing 0.5 mM ammonium as substrate. When ammonium was depleted, it was replenished to 400–500 μM with sterile 5 M NH_4_Cl solution. Ammonium measurements were done with Quantofix test stripes (Macherey-Nagel, Düren, Germany). The pH was manually adjusted to approx. 7.4 with 5% (w/v) NaHCO_3_ solution. For sample A4, 1 ml was inoculated in 100 ml mineral medium for NOB with 0.3 mM nitrite as substrate in a 250 ml screw cap Schott bottle. Both cultures were grown at 42°C under static conditions and evaporation was compensated for with sterile distilled water. Both cultures were regularly transferred with 1% (v/v) inoculum into mineral NOB media containing different concentrations of ammonium and nitrite with or without addition of 0.5 mM bicarbonate, and incubated under different conditions, as detailed in Supplementary Table S2.

### Isolation Attempts

Cells from the enrichment cultures Z2.3.3 and A4.5.1 (Supplementary Table S2) were manually separated by an optical tweezers system (PALM MicroTweezers, Carl Zeiss Microscopy GmbH, Munich, Germany). Cells were visualized by bright-field microscopy at 1000× magnification (Axio Observer.Z1, Carl Zeiss, Jena, Germany), trapped with an infrared laser (1064 nm, 3 W) and separated by micromanipulation (PatchMan NP2, CellTram Vario, Eppendorf, Hamburg, Germany). Separated cells were transferred to NOB medium (0.5 mM NaNO_2_, 0.5 mM NH_4_Cl and 0.5 mM NaHCO_3_) in 1.5 ml Eppendorf tubes, and incubated at 37°C. A total of 10 and 20 tubes for enrichments Z2.3.3 and A4.5.1, respectively, were inoculated with single cells or short chains resembling *Nitrolancea* by morphology. After nitrite consumption, active cultures were transferred twice to 5ml tubes (10% inoculum) and lastly into 100 ml Erlenmeyer flasks with 75 ml NOB medium. Additional attempts to eliminate accompanying heterotrophic bacteria were serial tenfold dilutions for cultures Z2.3.3 and A4.5.2 in three to four parallels in mineral NOB medium containing 0.5 mM NaNO_2_, 0.5 mM NH_4_Cl and 0.5 mM NaHCO_3_, and incubated at 37°C. All investigations of *Nitrolancea* were done on enrichment cultures.

### Purity Tests

The purity of the cultures (in terms of absence of heterotrophs) was tested repeatedly by incubating culture aliquots in complex liquid medium (0.5 g l^–1^ bactopeptone, 0.5 g l^–1^ yeast extract, 0.5 g l^–1^ meat extract, 0.584 g l^–1^ NaCl in distilled water, pH 7.4), and on solid RT (2.5 g l^–1^ meat extract, 2.5 g l^–1^ casamino acids, 0.5 g l^–1^ yeast extract, 1.0 g l^–1^ KH_2_PO_4_, 0.5 g l^–1^ NaCl, pH 7.4, 15.0 g l^–1^ agar in distilled water), R2A (half-strength; 0.25 g l^–1^ yeast extract, 0.25 g l^–1^ proteose peptone, 0.25 g l^–1^ casamino acids, 0.25 g l^–1^ glucose, 0.25 g l^–1^ soluble starch, 0.25 g l^–1^ Na-pyruvate, 0.15 g l^–1^ K_2_HPO_4_, 0.025 g l^–1^ MgSO_4_ × 7 H_2_O, pH 7.4, 15.0 g l^–1^ agar in distilled water) or mixotrophic NOB medium (0.2 g l^–1^ NaNO_2_, 0.15 g l^–1^ yeast extract, 0.15 g l^–1^ bactopeptone, 0.055 g l^–1^ sodium pyruvate, trace elements [see mineral medium above], pH 8.0, 13 g l^–1^ agarose in distilled water).

### Physiological Experiments

Growth of *Nitrolancea* was investigated in batch cultures, using 100 ml mineral medium in 300 ml Erlenmeyer flasks under static conditions at 37°C. Cultures were inoculated with 1% (v/v). To test for nitrite consumption as indication for growth, the Griess-Ilosvay spot test was used (Schmidt and Belser, 1994) or nitrite and nitrate were determined qualitatively with analytical test stripes (Merck KGaA, Germany). Nitrite concentrations for physiological experiments were measured by the HPLC technique.

### Chemical Analyses

Nitrite and nitrate were determined quantitatively by HPLC via ion pair chromatography on a LiChrospher RP-18 column (125 × 4 mm; Merck) with UV detection in an automated system (Hitachi LaChrom Elite; VWR International GmbH, Darmstadt, Germany). Data acquisition and processing of nitrite and nitrate concentrations was performed with the integrated software EZChrom Elite 3.3.2.

### Growth Characteristics

Nitrite consumption, nitrate production, cell numbers and protein content of cultures of A4.5.2 and Z2.3.4 were determined to calculate generation times (d^–1^) as well as protein and cell yield per mmol nitrite. Cells were counted using a Thoma cell chamber (0.02 mm depth), and protein content was quantified by fluorescence using the NanoOrange protein quantitation kit (Thermo Fisher Scientific, Waltham, MA, United States).

### Activity Measurements and Oxidation Kinetics

Nitrite-dependent oxygen consumption of cells from culture of A4.5.2 (1 replicate) and culture Z2.3.4 (2 biological replicates) was measured following Nowka et al. (2015), using a micro-respiration system (Unisense AS, Denmark) consisting of a 1-channel oxygen sensor amplifier (OXY-Meter), a Clark-type oxygen microsensor (OX-MR), 2 ml glass chambers with glass stoppers, glass coated magnets and a stirring system. Cells were grown at 37°C in mineral medium as described above, with 10 mM and 35 mM (A4.5.2) or 5 mM and 15 mM (Z2.3.4) ammonium and nitrite, respectively. 12–48 h after nitrite was depleted, cells were added to the chambers, which were sealed and mounted on a stirring rack in a water bath at 37°C. The oxygen microsensor was inserted through a capillary hole inside the glass stopper. After an initial equilibration period of 15–30 min, the measurements were started by adding nitrite from stock solutions through a second capillary hole using a syringe. Nitrite oxidation kinetics were estimated from oxygen consumption rates at varying defined nitrite concentrations using the R-package “drc” (Ritz et al., 2015), which fits Michaelis-Menten kinetic to the data with the following equation: *V* = (V_max_ × [S])/(K_m_ + [S]), where V is activity, V_max_ is maximum specific activity (μmol mg protein^–1^ h^–1^), K_m_ is the half-saturation constant for nitrite oxidation (μM), and [S] is concentration of nitrite (μM). For cell specific V_max_, the calculated maximum activity was translated to fmol NO_2_^–^ cell^–1^ h^–1^.

### Microscopic Investigations

For electron microscopy, cells were collected by centrifugation (13,000 × *g* for 15 min), fixed with 2.5% (v/v) glutaraldehyde as well as 2% (w/v) osmium tetroxide, and embedded in a mixture of Spurr and acetone as described previously (Spieck and Lipski, 2011). Thin sections were stained with 2% (w/v) uranyl acetate and 2% (w/v) lead citrate. Microscopic examination was carried out with a transmission electron microscope (Zeiss model Leo 906E with a CCD camera model 794). For the visualization of whole cells, 3 μl concentrated biomass was pipetted on EM grids (300 mesh, Stork Veco B.V, Netherlands) and stained with 2% (w/v) uranyl acetate. 4′-6′-diamidino-2-phenylindole (DAPI) stained cells were observed using a confocal laser scanning microscope LSM 800 (Zeiss, Jena, Germany) and Plan-Apochromat 63x and 100 × 1.4 oil objectives.

### Fluorescence *in situ* Hybridization

Cells were pelleted at 10°C and 13,000 × *g* for 15 min, washed with 0.9% (w/v) NaCl and fixed with 1:1 (v/v) 96% ethanol:PBS as described previously (Amann et al., 1995). After dehydration in 50, 80 and 96% ethanol for 3 min each (Manz et al., 1992), cells were hybridized overnight in hybridization buffer containing 20% formamide with the FITC-labeled universal bacterial probe EUB338 (Amann et al., 1990) and the Cy3-labeled probe Ntlc804 specific for *Nitrolancea* (Sorokin et al., 2012). Cells were embedded in Citifluor AF1 (Citifluor Ltd., London, United Kingdom) prior to microscopic observation at the LSM 800 (Zeiss). Signal specificity was ensured by performing negative control hybridizations using the NON-338 probe labeled in Cy3 (Wallner et al., 1993).

### DNA Extraction

DNA for PCR and Illumina sequencing was extracted with the PowerSoil DNA isolation Kit (MO BIO Laboratories, Inc., Carlsbad, CA, United States) according to manufacturer’s instructions with slight modifications except for cultures E2 and A4.1: Before cell disruption via vortexing (step 5), Proteinase K (1 mg ml^–1^), lysozyme (4 mg ml^–1^) and RNase A (1 mg ml^–1^) were added. The vortexing step was extended to 30 min at 37°C.

### PCR

The presence of *Nitrolancea* was tested for with specific primer sets for 16S rRNA and *nxrA* genes (Supplementary Table S3) and subsequent gel electrophoresis. Presence of NOB belonging to *Nitrospira* was checked using the *nxrB*-targeted primer set NxrB-169f/NxrB-638r (Pester et al., 2014). The following PCR program was used: initial denaturation at 94°C, 5 min; 32 cycles consisting of 30 s denaturation at 94°C, 30 s annealing at 55°C (16S rRNA gene), 60°C (*nxrA*) or 56°C (*nxrB*) and 3.5 min (for *Nitrolancea* primers) or 45 s (*Nitrospira* primers) elongation at 72°C; final elongation at 72°C, 20 min.

### Amplicon Sequencing

For selected cultures, 10 ng of genomic DNA were used for 16S rRNA gene amplicon sequencing at MR DNA (Molecular Research LP, Shallowater, TX, United States). For the cultures E2 and A4.1, amplicons were generated using the primers 341F and 785R (Herlemann et al., 2011), followed by 454 pyrosequencing (300 bp, 3000 reads per sample). For all other cultures the primers 515F and 806R (Caporaso et al., 2011) were used with subsequent Illumina MiSeq sequencing (2 × 300 bp, 20000 reads per sample). Operational taxonomic units (OTUs) were obtained from the preprocessed sequences using the *de novo* OTU picking workflow in qiime (v1.9.1) (Caporaso et al., 2010), and summarized and visualized by the QIIME *summarize_taxa_through_plots.py* script. Illumina sequences were processed, classified and summarized by MR DNA (Dowd et al., 2008). In short, paired-end sequences were joined and depleted of barcodes, followed by removal of sequences <150 bp or with ambiguous base calls. Sequences were denoised, OTUs generated and chimeras removed. OTUs were defined by clustering at 3% divergence (97% similarity). Final OTUs were taxonomically classified using BLASTn against a curated database derived from NCBI and RDPII.^1^,^2^

### Metagenomic Sequencing

DNA was fragmented using a Bioruptor (Diagenode, Seraing, Belgium). Libraries for Illumina HiSeq sequencing were generated using the NEBNext Ultra DNA Library Prep Kit for Illumina (New England Biolabs, Ipswich, MA, United States) as per manufacturer’s recommendations. Size and quality of the libraries were visualized on a Bioanalyzer High Sensitivity Chip (Agilent Technologies, Santa Clara, CA, United States). Diluted libraries were multiplex sequenced on the Illumina HiSeq 2500 instrument (Illumina, St. Diego, CA, United States) by paired-end sequencing (2 × 100 bp [metagenomes 40 and 51] or 2 × 250 bp [metagenome 35] on the Illumina MiSeq platform (Illumina, St. Diego, CA, United States). For sequencing of the metagenomes A2, Z2, and Z4, library preparation was done using the Nextera XT kit (Illumina, San Diego, CA, United States) according the manufacturer’s instructions. Tagmentation was performed starting with 1 ng of DNA, followed by incorporation of the indexed adapters and amplification of the library. After purification of the amplified library using AMPure XP beads (Beckman Coulter, Indianapolis, IA, United States), libraries were checked for quality and size distribution using the Agilent 2100 Bioanalyzer and the High sensitivity DNA kit. Quantitation of the library was performed by Qubit with the dsDNA HS Assay Kit (Thermo Fisher Scientific Inc., Waltham, MA, United States). The libraries were pooled, denatured and sequenced on a Illumina MiSeq (San Diego, CA, United States). Paired end sequencing of 2 × 300 bp was performed using MiSeq Reagent Kit v3 (San Diego, CA, United States) according to the manufacturer’s protocol.

### Metagenome Assembly and Binning

Adapter removal, contaminant filtering and quality trimming of the Illumina HiSeq and MiSeq paired-end sequencing reads was performed using BBDUK (BBTOOLS version 37.76). Terminal base calls with a quality score < Q17 were trimmed and the reads with a mean quality score of ≤Q20 were discarded. The processed HiSeq reads with a length of ≥70 bp were then corrected for sequencing errors using BayesHammer (Nikolenko et al., 2013). Additional filtering parameters were applied to the MiSeq reads (*k* = 23, mink = 11, hdist = 1, ktrim = r, tbo = t, maxns = 0, tossjunk = t) and only reads with a length of ≥150 bp were kept. The corrected reads were assembled using metaSPAdes v3.11.1 (Nurk et al., 2017) with default settings. For the samples sequenced with HiSeq, the reads for all samples were co-assembled with iterative assemblies using kmer sizes of 21, 33, 55, 77, 99, and 127, whereas for the MiSeq reads, the assemblies were performed separately for each sample with kmer sizes of 21, 33, and 55. Trimmed HiSeq reads were mapped back to the metagenome separately for each sample using Burrows-Wheeler Aligner (BWA v0.7.17) (Li and Durbin, 2009), employing the “mem” algorithm. For samples sequenced with MiSeq, reads were mapped back to the metagenomes using the minimap2 (v2.16-r922) preset for genomic short-read mapping (Li, 2018). The sequence mapping files were handled and converted as needed using SAMtools 1.6 (Li et al., 2009). The MiSeq metagenomes were manually binned using anvi’o (v5, Eren et al., 2015) and taxonomically classified using the ‘classify workflow’ of GTDB-Tk (version 0.3.2, Parks et al., 2018). Subsequently, the *Nitrolancea*-like bins were annotated using Prokka (version 1.12-beta, Seemann, 2014) with the RefSeq bacterial nr protein database as a reference and the Kyoto Encyclopedia of Genes and Genomes (KEGG). The completeness and redundancy of the bins was assessed using the checkM ‘lineage’ workflow (Parks et al., 2015).

### Phylogenetic Analyses

The average nucleotide identity (ANI) between the recovered *Nitrolancea* genomes and the genome of *Nl. hollandica* was calculated using orthoANI (Lee et al., 2016).

Sequences (16S rRNA and *nxrA*) were aligned using the multiple sequence alignment web server T-Coffee (Notredame et al., 2000). Subsequently, maximum-likelihood trees were calculated using Mega-X (Kumar et al., 2018) with 1000 bootstraps.

### Genome Comparison

The orthologous gene clusters were identified using the OrthoVenn 2 web service tool (Xu et al., 2019) with an *E*-Value of 1e-2 and an inflation value of 1.5.

## Results

### Selective Enrichment of Nitrolancea

The enrichment process of *Nitrolancea* from WWTP centrate and geothermal mud pool samples was monitored by specific PCR, fluorescence *in situ* hybridization (FISH; Supplementary Table S3), and 16S rRNA gene amplicon and metagenomic sequencing. Additional details on the enrichment process can be found in the Supplementary Material.

As shown by amplicon sequencing, the microbial community of the original centrate treatment reactor sample Z2 was dominated by *Proteobacteria* (56%), *Bacteriodetes* (22%) and *Firmicutes* (10%). *Chloroflexi* were present at low amounts (2%; Supplementary Figure S2), but none of these sequences were affiliated with the genus *Nitrolancea*. Members of the nitrifying genera *Nitrosomonas* and *Nitrosovibrio* were detected (2.1 and 0.7%, respectively), but no known nitrite oxidizer could be identified.

**FIGURE 1:**
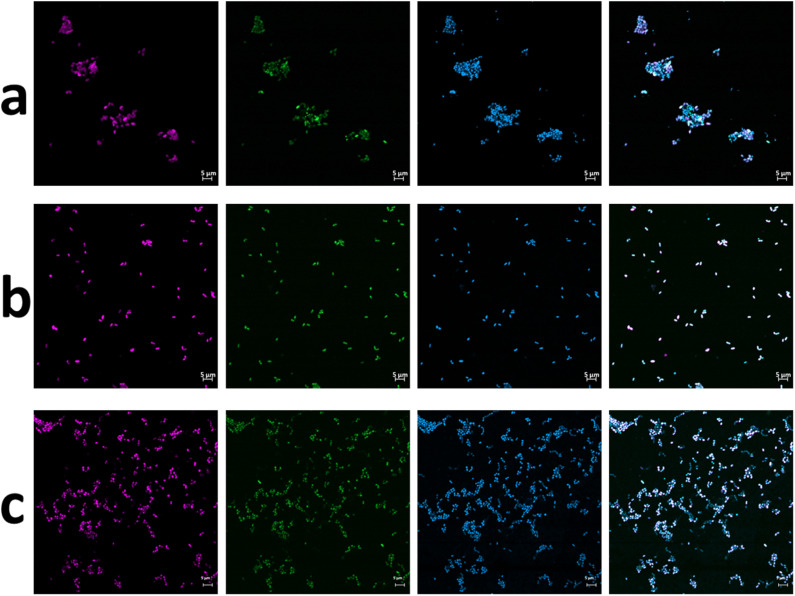
FISH micrographs of culture Z2.3.3 **(a)** before and **(b)** after 10^−8^ dilution and **(c)** of culture A4.5.2. From left to right: *Nitrolancea*-like bacteria detected with probe Ntlc804 labeled in Cy3 (red); all bacteria detected with EUB338 labeled in FITC (green); all cells stained with DAPI (blue); overlay of all three channels.

Over a period of only 4 months, *Nitrolancea* could be selectively enriched from 1.4 to 24% (Supplementary Table S2). Different isolation attempts were performed to increase the relative abundance of *Nitrolancea*-like bacteria in the nitrite-oxidizing cultures. For Z2, incubations with increasing substrate concentrations (up to 70 mM nitrite) revealed a very high enrichment of lancet-shaped cells (Figure 1a) and combination with the use of an optical tweezers system or serial dilution series increased the enrichment further (Figure 1b). Two consecutive dilution series with growth up to the dilution 10^–8^ and 10^–6^, respectively, lead to a pure culture of *Nitrolancea* strain Z based on purity tests using four different complex media.

Unlike the centrate treatment reactor sample, the primary nitrite-oxidizing enrichment of Las Máquinas (A4.1) grown at 42°C was dominated by members of the phylum *Chloroflexi* (45%), followed by *Deinococcus-Thermus* (28%) and *Proteobacteria* (22%) according to 16S rRNA amplicon sequencing (Supplementary Figure S2). This enrichment had been supplied with 0.3 mM of nitrite without ammonium addition. *Nitrolancea* abundances increased to 14.1% after the culture had been repeatedly replenished with nitrite over a period of 4 years and increased further to 44% after using high substrate concentrations (30 mM) and adding ammonium (Supplementary Table S2). Manual sorting of cells from this enrichment with the optical tweezers system generated six follow-up cultures that, however, all still contained several accompanying heterotrophic bacteria (not shown). Meanwhile, the parallel culture A4.5.2, which received 40 mM nitrite in addition to the ammonium and bicarbonate also present in A4.5.1, was nearly pure as shown by inoculation of four different complex media and FISH (Figure 1c) and only tiny colonies grew on half-strength R2A plates. These heterotrophic bacteria were lost upon another serial dilution to the step 10^–7^ (A4.5.3) and the strain deemed isolated. The novel *Nitrolancea* strain was provisionally designated as *Ca.* Nitrolancea copahuensis based on 16S rRNA gene and whole-genome dissimilarity to *Nl. hollandica* (see below).

Notably, in an ammonia-oxidizing culture that had been inoculated with an earlier sample from Las Maquinas (E2) with 0.5 mM ammonium, but without nitrite addition, *Nitrolancea*-like NOB were also co-enriched with unknown ammonia-oxidizing microorganisms. This culture was dominated by *Chloroflexi* (34%), *Proteobacteria* (40%) and *Actinobacteria* (21%), while *Nitrolancea*-like bacteria constituted nearly 20% of the microbial community after 3.5 years of incubation at 42°C. Contrastingly, no *Nitrolancea-*like sequences could be detected in a parallel nitrite-oxidizing culture of E2, which was grown without ammonium addition at 42°C (not shown).

Regarding other known NOB, *Nitrospira* were nearly absent in all centrate treatment reactor enrichments and constituted only 0.3% of the retrieved amplicon sequences in the Argentinian cultures A4.2, A4.3 and A4.4 (Supplementary Table S2). *Nitrobacter*-like 16S rRNA gene sequences amounted to 0.8% in the centrate cultures Z2.1 and Z2.2 (not shown). In the Argentinian A4 cultures, *Nitrobacter* was undetectable, but constituted a tiny fraction in culture E2 (0.05%).

### Morphology and Ultrastructure

**FIGURE 2:**
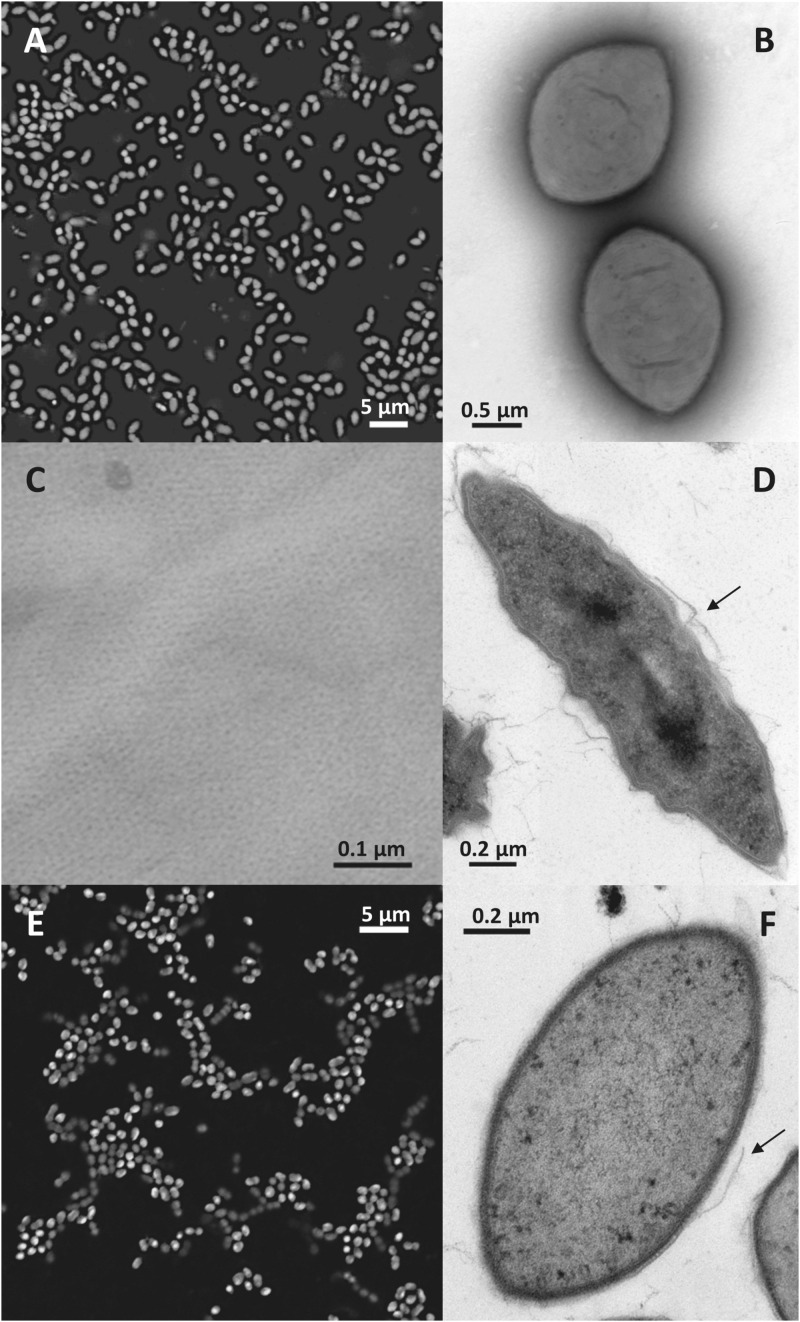
*Nitrolancea*-like cells in the nitrite-oxidizing enrichments derived from centrate **(A–D**; culture Z2.3.3) and of Las Máquinas **(E,F)**. **(A)** Cell shape of *Nitrolancea hollandica* strain Z stained with DAPI. **(B)** Negatively stained cells showing the tapered ends in detail, stained with uranyl acetate. **(C)** Regular protein layer of the cell wall. **(D)** Electron micrograph of an ultrathin section. **(E)** Fluorescence micrograph of culture A4.4 stained with DAPI. **(F)** Ultrathin section of culture A4.3. The arrows indicate where the disrupted external protein layer is detached from the cell wall.

Light and transmission electron microscopy of the nitrite-oxidizing enrichment cultures that had been inoculated with biomass from the Hamburg-Dradenau centrate treatment reactor and from Las Máquinas revealed *Nitrolancea*-like cells with characteristic tapered ends (Figure 2) similar to those of *Nl. hollandica* (Sorokin et al., 2012), but smaller in size (Table 1). The lancet shape was less pronounced in A4 cultures, where most cells had an ovoid shape (Figure 2F). *Nitrolancea*-like bacteria in all cultures appeared as single cells, or formed pairs and short chains. In late nitrite-oxidizing cultures, cells of *Nitrolancea* aggregated to roundish flocs, whereas in primary enrichments of cultures E2 and A4 tiny yellow to orange particles accumulated.

**TABLE 1:** Comparison of morphological, physiological and genomic features of the novel *Nitrolancea* strains and *Nl. hollandica.*

**Feature**	*Nl. hollandica* strain Z*	***Ca.* Nl. copahuensis**	***Nl. hollandica***
Morphology	Ovoid rods with tapered ends	Ovoid rods with tapered ends	Ovoid rods with tapered ends
Size [μm]	1.3–1.5 × 1.6–2.3	1.1 × 1.7	1–1.2 × 2–4
Temperature optimum	37°C	35–37°C	40°C
Temperature range for growth	19–45°C	19–45°C	20–46°C
Nitrite tolerance	150 mM	40 mM	75 mM
Nitrite optimum	3–10 mM	3–10 mM	5–20 mM
Km value (nitrite)	0.12–0.45 mM	0.22 mM	1 mM
μ_max_ (nitrite) h^–1^	0.013	0.016	0.019
NxrA copies	2/4 (Z2/Z4)	2	4
Ammonium requirement	+	+	+
Ammonium optimum	5–20 mM	5–25 mM	5 mM
Ammonium tolerance	500 mM	500 mM	>200 mM
CO_2_ fixation	Calvin cycle	Calvin cycle	Calvin cycle
Rubisco	Type I	Type I	Type I
Isolation source	Centrate treatment reactor	Geothermal spring	Bioreactor

*Without differentiation between the genotypes Z2 and Z4.

The *Nitrolancea*-like cells in both incubation series had the typical Gram-positive type complex cell wall, including a regular proteinaceous surface layer (Figure 2C), which is a common component of archaeal and bacterial cell walls (Rachel et al., 1997). This protein layer is easily disrupted during the embedding process (Figures 2D,F), resulting in loss of the typical cell morphology. As described for *Nl. hollandica*, the novel *Nitrolancea* strains did not possess carboxysomes or intracytoplasmic membrane systems. However, in some cells membrane invaginations as described by Sorokin et al. (2014) were observed at the cell poles (not shown).

### Physiology

**FIGURE 3:**
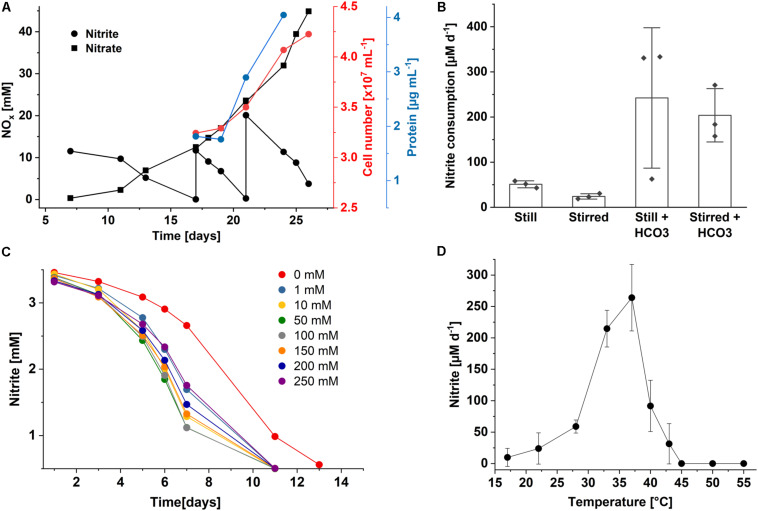
Growth characteristics of *Nitrolancea hollandica* strain Z. **(A)** Stoichiometric oxidation of nitrite to nitrate leading to increase in cell number and biomass. **(B)** Nitrite consumption of enrichment culture Z2.3.3 under different incubation conditions at 37°C: without agitation (still) or stirred at approx. 120 rpm, and with or without the addition of 0.5 mM NaHCO_3_. Bars represents the mean ± standard error of biological triplicates, diamonds indicate individual data points; **(C)** nitrite oxidation over time in medium supplemented with 3 mM nitrite and different ammonium concentrations (0 – 250 mM); **(D)** nitrite oxidation rates per day at different temperatures. The rates were determined for the period between day 3 and 10 after inoculation (2.5 mM initial nitrite concentration). Data represents the mean ± standard error of biological triplicates. For all incubations, medium was inoculated with 1% (v/v) enrichment culture of **(A)** Z2.3.4, **(B,D)** Z2.3.3 or **(C)** Z2.3.1.

**FIGURE 4:**
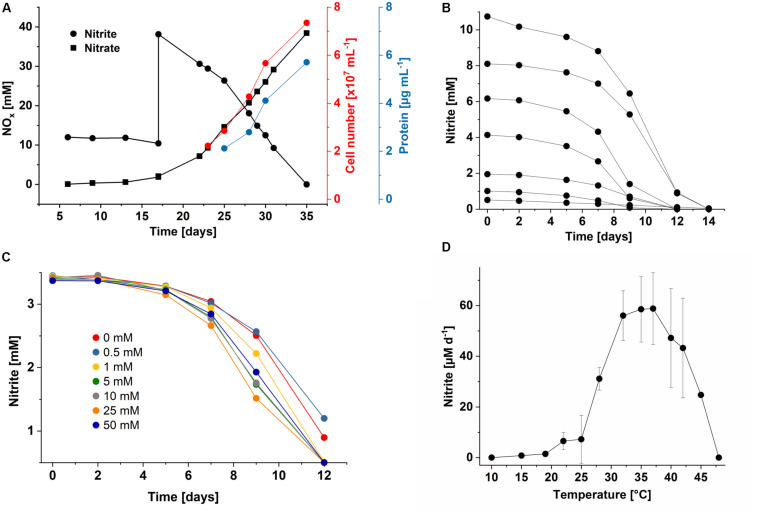
Growth characteristics of *Ca.* Nitrolancea copahuensis. **(A)** Stoichiometric oxidation of nitrite to nitrate leading to increase in cell number and biomass. **(B)** Substrate consumption test with different initial nitrite concentrations in culture A4.4, showing the rapid consumption of nitrite. Reduced nitrite oxidation was still observed at up to 40 mM nitrite (not shown). **(C)** Nitrite consumption of culture A4.4 in medium supplemented with different ammonium concentrations (0 to 50 mM). **(D)** Influence of temperature on nitrite oxidation in culture A4.5.1 with 0.5 mM nitrite. Nitrite oxidation rates were determined for the period between day 4 and 11 after inoculation. Data represents the mean ± standard error of biological triplicates. At 42°C, two biological replicates were incubated.

As also known for *Nl. hollandica*, cells of both *Nitrolancea* enrichments derived from the samples Z2 and A4 were able to grow lithoautotrophically with nitrite as energy source and CO_2_ as carbon source. Nitrite was oxidized aerobically and stoichiometrically to nitrate with a minimum generation time of 53 h (culture Z2.3.4; Figure 3A) or 43 h (culture A4.5.2; Figure 4A).

In the beginning of the cultivation process, the Z2.2 culture was tested for its ammonium requirements. Growth was slow in ammonium-free medium and was clearly accelerated by the addition of 0.5 – 3 mM ammonium (not shown). Consequently, all mineral NOB media were prepared with the addition of ammonium. Fascinatingly, NH_4_Cl concentrations up to 500 mM could be tolerated, although consumption of 3 mM nitrite took 4 months. Addition of 250 mM ammonium caused slight inhibition only (Figure 3C). In accordance with *Nl. hollandica*, the cultures derived from the centrate were able to grow in the presence of very high nitrite concentrations and completely oxidized up to 150 mM nitrite within several months. Growth occurred between 22 and 43°C and with a delay at 19 and 45°C. Nitrite was oxidized optimally at 37°C (Figure 3D). Additionally, the influence of aeration and bicarbonate supplementation on growth and activity were tested. The highest nitrite oxidation rates were obtained when cells were incubated in Erlenmeyer flasks without agitation and were further accelerated by addition of 0.5 mM NaHCO_3_ (Figure 3B).

The nitrite oxidation rates of the *Nitrolancea* cultures obtained from Las Máquinas (A4.4) increased with increasing substrate concentrations (1–10 mM NaNO_2_) without a prolonged lag-phase (Figure 4B). The nitrite tolerance level was 40 mM. Nitrite was also oxidized slowly in medium without ammonium, but optimal growth depended on the addition of up to 50 mM NH_4_Cl (Figure 4C). The ammonium tolerance limit was 500 mM. Growth of the Argentinian *Nitrolancea* strain was observed between 22 and 45°C, delayed at 19°C, and with an optimum growth temperature of 35–37°C (Figure 4D). A comparison of key parameters between *Nl. hollandica* and the strains investigated here is shown in Table 1.

Nitrite oxidation kinetics were determined by microsensor-based oxygen consumption measurements in the different *Nitrolancea* cultures (Supplementary Figure S3). Both representatives revealed moderate substrate affinities with apparent half-saturation constants (K_m_) of 0.12–0.45 mM nitrite for *Nl. hollandica* strain Z and 0.22 mM for *Nl. copahuensis* (Supplementary Table S4). The maximum specific activity (V_max_) was relatively low (11.1–26.8 μmol nitrite mg protein^–1^ h^–1^). Growth yields ranged from 0.17 to 0.88 mg protein mmol nitrite^–1^.

### Genomic Analyses

Biomass of cultures Z2.2, Z2.3.2, and A4.4 was used for metagenomic sequencing (Table 2). The draft genomes of the three resulting *Nitrolancea* strains described here (Z2, Z4, A2) could be assembled into 48 to 150 contigs. These high-quality draft genomes ranged in size from 3.19 to 3.45 Mbp and had G+C contents between 62.7 and 62.9 mol%. The number of predicted coding sequences was between 3023 and 3235, including 40 to 46 tRNAs.

**TABLE 2:** Genome characteristics of the four *Nitrolancea* strains as predicted using PROKKA (Version 1.12).

**Genomic feature**	***Nl. hollandica* Z2**	***Nl. hollandica* Z4**	***Ca.* Nl. copahuensis A2**	***Nl hollandica* Lb**
Genome size	3 193 324 bp	3 450 053 bp	3 385 528 bp	3 888 104 bp
G+C value (genome)	62.9%	62.9%	62.7%	62.6%
CDS	3023	3235	3232	3946
Contigs	150	140	48	736
tRNA	41	40	46	67
tmRNA	1	1	1	1
Completeness	99.7%	98.1%	96.3%	
Contamination	0%	0%	0%	

**FIGURE 5:**
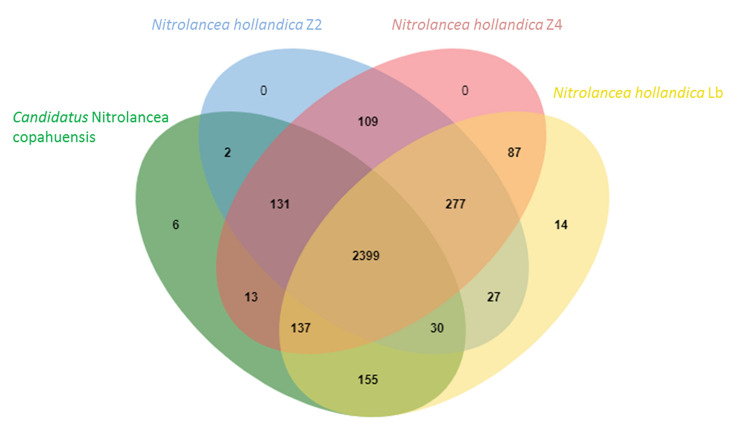
Venn diagram indicating the number of shared orthologous clusters among the genomes of *Nitrolancea hollandica* and the *Nitrolancea* strains obtained from the nitrite-oxidizing enrichments A4.4 (A2), Z2.2 (Z2) and Z2.3.2 (Z4). The diagram was plotted by using OrthoVenn2 (Xu et al., 2019).

After assembly and binning of the *Nitrolancea* strains, comparative genomics revealed that two highly similar strains (Z2 and Z4) were enriched from the centrate treatment reactor sample, while the strain A2 obtained from sample A4 was clearly distinct (see below). The comparison of these metagenome-assembled genomes (MAGs) with *Nl. hollandica* revealed 2399 conserved clusters out of in total 3387 orthologous gene clusters within the four *Nitrolancea* strains (Figure 5). The type strain *Nl. hollandica* exhibits 14 unique gene clusters that were absent in the genomes of the three novel strains, which, on the contrary, shared 131 of orthologous clusters. Additionally, the Argentinian strain A2 contained 6 unique gene clusters, while the two genomes obtained from the centrate enrichment cultures in total had 109 clusters not present in the other *Nitrolancea* strains.

**FIGURE 6:**
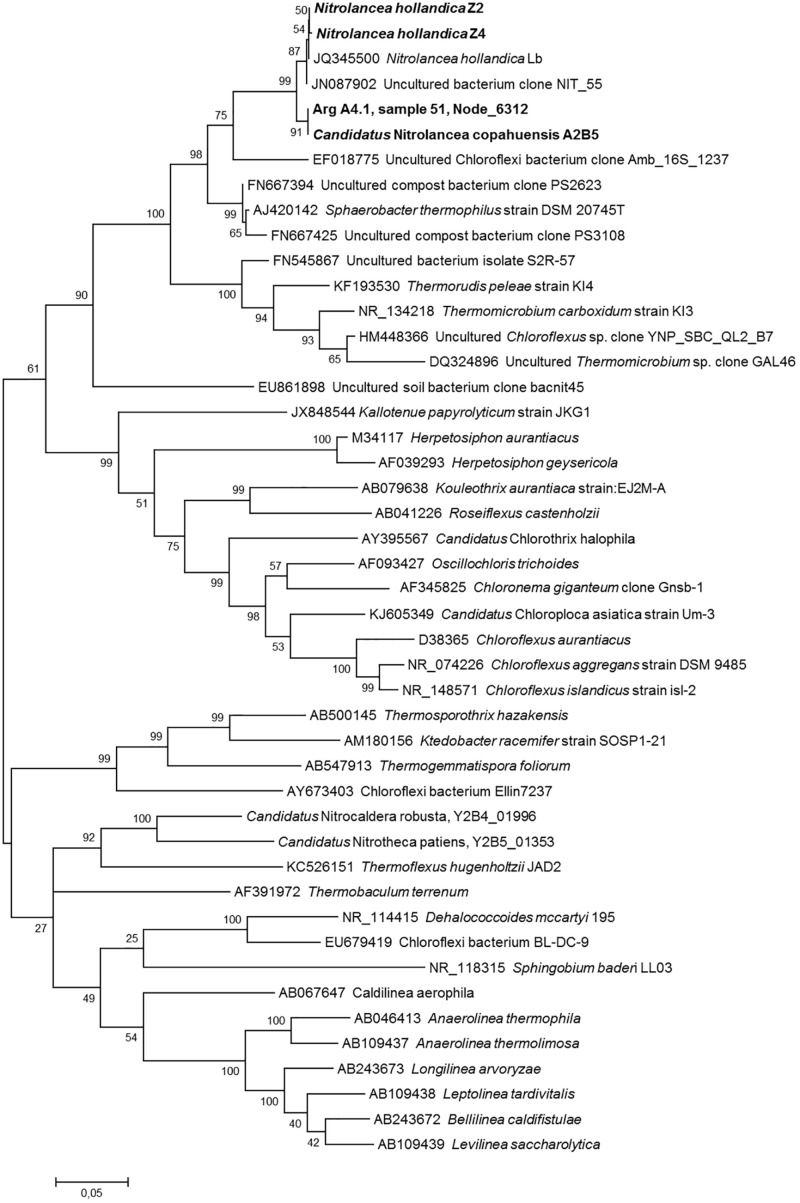
Phylogenetic relationship of the *Nitrolancea* strains based on 16S rRNA gene sequences. Maximum likelihood tree indicating the affiliation of the 16S rRNA genes identified in the centrate treatment reactor and Argentinian enrichment cultures. Statistical branching support values based on 1000 bootstraps are indicated on the nodes. The alignment contained 1703 valid positions used for tree calculation. Sequences obtained in this study are shown in bold. The scale bar represents the expected changes per nucleotide. For designation of cultures see Supplementary Table S2.

Phylogenetic and sequence similarity-based analysis of the 16S rRNA genes of the novel *Nitrolancea* strains (Supplementary Table S5, Figure 6) revealed a close affiliation with the previously described type strain *Nl. hollandica* (Sorokin et al., 2014). *Ca.* Nitrolancea copahuensis had a 16S rRNA gene sequence identity of 98.5% to *Nl. hollandica*, whereas the two centrate strains were almost identical to the type strain (99.9 and 100% identity). Comparison of the NxrA sequences of the novel strains and *Nl. hollandica* revealed high amino acid identities (Supplementary Table S5, Supplementary Figure S4). Furthermore, the draft genome sequences of the centrate strains and *Nl. hollandica* had high average nucleotide identity (ANI) values of 99.2 and 99.3%, respectively, which was only 91.9% for the geothermal strain (Supplementary Table S6). The two draft genomes from the centrate enrichments were almost identical and had an ANI value of 99.96%. Thus, based on 16S rRNA gene and ANI similarity, Z2 and Z4 belong to the species *Nl. hollandica*, while the Argentinian strain A2 had 16S rRNA and ANI identity values below the species threshold of 98.7–99.0% (Stackebrandt and Ebers, 2006) and 95% (Rodriguez-R and Konstantinidis, 2016), respectively. Consequently, this strain represents a separate species within the genus *Nitrolancea*.

The genomic data was furthermore mined to reconstruct the core metabolic pathways of the three novel *Nitrolancea* strains (Table 3). Genes encoding all proteins of the Calvin cycle for CO_2_ fixation, but not for carboxysomes have been identified. The potential for nitrite and formate oxidation was also present and the genomes contained additional non-operonal *nxrA* copies, as has been observed for *Nl. hollandica* (Sorokin et al., 2012). The A2 and Z2 genomes contained only one orphan *nxrA* gene, while the Z4 genome encoded 3 additional NxrA copies. All other NXR subunits were not duplicated and encoded within a highly conserved operon (Supplementary Figure S5). Furthermore, genes for a NO-forming nitrite reductase (*narK*) and the cytoplasmic assimilatory nitrate reductase (*nasAB*) were detected in the three analyzed genomes, but not for assimilatory nitrite reductases (*nirA or nirBD*) or dissimilatory nitrite reduction to ammonium (*nrfA*).

**TABLE 3:** Comparison of key functions predicted for the four *Nitrolancea* strains using PROKKA (Version 1.12) and the Kyoto Encyclopedia of Genes and Genomes (KEGG).

**Predicted function**	***Nitrolancea hollandica***	***Ca.* Nl. copahuensis A2**	***Nl. hollandica* Z2**	***Nl. hollandica* Z4**
Heterotrophy	yes*	yes	yes	yes
Carbon fixation	Calvin cycle	Calvin cycle	Calvin cycle	Calvin cycle
Carbon monoxide dehydrogenase	yes*	yes	yes	yes
Formate dehydrogenase	yes	yes	yes	yes
Hydrogenase	yes	yes	not complete	yes
Nitrite oxidation	yes	yes	yes	yes
Assimilatory nitrite reduction	no	no	no	no
Assimilatory nitrate reduction	yes	yes	yes	yes
Sulfite oxidation	no	no	no	no
Urea degradation	no	no	no	no
Cyanate degradation	yes	yes	yes	yes
Siderophore transport	yes	yes	no	yes
Flagellum	no	no	no	no
Secretion system	Sec + Tat	Sec + Tat	Sec + Tat	Sec + Tat

*Could not be functionally confirmed (Sorokin et al., 2012).

Additionally, all *Nitrolancea* genomes encoded an aerobic-type CO dehydrogenase, cyanase and an [NiFe]-hydrogenase. Based on the genomic data, all strains have the potential to grow heterotrophically and to use thiosulfate for energy conservation. So far, none of these phenotypes could be observed in growth experiments with *Nl. hollandica* (Sorokin et al., 2012). Within the sulfur metabolism, the assimilatory sulfate reduction pathway involving sulfate adenylyltransferase (*sat*), adenylyl-sulfate kinase (*cysC*), phosphoadenosine phosphosulfate reductase (*cysH*) and ferredoxin-dependent sulfite reductase (*sir*) could be identified in the three analyzed genomes, along with genes encoding thiosulfate sulfurtransferases (*glpE*, *sseA*) and a putative sulfite oxidase (*sorA*).

## Discussion

The first isolate of *Nitrolancea* was only described in 2012 (Sorokin et al., 2012). Growth depended on the use of a modified NOB medium (Spieck and Lipski, 2011), as *Nl. hollandica* grew optimally in mineral medium containing high substrate concentrations of 20 to 50 mM KNO_2_ and supplementation of 5 mM NH_4_HCO_3_. Unexpectedly for a nitrite-oxidizing organism, *Nl. hollandica* depends on ammonium as N-source and accordingly its genome did not encode any enzymes for nitrite reduction to ammonium (Sorokin et al., 2012). This absence of assimilatory nitrite reduction pathways was recently also observed in other thermophilic NOB within the phylum *Chloroflexi* (Spieck et al., 2020), and some *Nitrotoga* species are apparently dependent on ammonium addition, too (Ishii et al., 2017; Wegen et al., 2019). In contrast, the early *Nitrolancea* enrichments obtained in this study were able to oxidize nitrite also without ammonium addition, although no assimilatory nitrite reductase genes were detected in their genomes. However, it is likely that these *Nitrolancea* strains were provided with ammonium or organic N-sources by the accompanying heterotrophs present in the cultures, as the ability for growth without ammonium addition was lost upon increased purity of the cultures.

The optimal growth conditions for the novel *Nitrolancea* strains were assessed by testing different incubation temperatures, as well as nitrite and ammonium concentrations. Although *Nitrolancea* has been described as NOB, that requires high substrate concentrations, early enrichments from Las Máquinas were done at very low nitrite availability (Supplementary Table S2). Nevertheless, once an active culture was obtained, an increase of substrate concentrations was observed to be beneficial for the selective enrichment of *Nitrolancea*-like NOB. High ammonium concentrations can also enhance proliferation of *Nitrolancea*, as was observed in culture A4.4. However, we cannot exclude that this effect was caused by growth stimulation of accompanying bacteria. After initial cultivation at 42°C, reducing the incubation temperatures to the apparent optimum of 37°C led to a reliable enrichment of *Nitrolancea*-like cells from both sample locations (Supplementary Table S2). Intriguingly, this cultivation strategy resulted in the successful enrichment of two novel *Nl. hollandica* strains from the centrate treatment reactor, even though no *Nitrolancea*-like 16S rRNA gene sequences were detected in the original sample. This finding indicates that these NOB are more widely distributed than previously assumed, albeit at very low numbers. The minimum temperature for growth for *Nitrolancea* derived from the centrate reactor as well as from the geothermal mud pool was 19°C, but related 16S rRNA gene sequences were also found in a bioreactor system operated at 12°C (Yu et al., 2018). Therefore, the environmental distribution of these NOB seems not to be restricted to thermophilic conditions.

Fast and reproducible initial enrichment of *Nitrolancea* spp. was achieved by designing a standard medium consisting of a mineral salt solution supplemented with 3 mM NaNO_2_, 3 mM NH_4_Cl and, as observed in later cultivation approaches, 0.5 mM NaHCO_3_. The positive effect of bicarbonate probably is due to resulting elevated CO_2_ concentrations within the cell, which might reduce the reaction of ribulose 1,5-bisphosphate carboxylase/oxygenase (RuBisCO) with the competing substrate O_2_ and thus increase carbon fixation efficiency in the absence of other CO_2_-concentrating mechanisms like carboxysomes (Price et al., 2008). In this study, a preference for a reduced oxygen supply (Figure 3B) of *N. hollandica* strain Z was found and culturing was done without agitation. Accordingly, *Nitrolancea*-like sequences have been detected in a reactor treating synthetic low-strength wastewater at low dissolved oxygen contents (Yu et al., 2020).

The proliferation of the different *Nitrolancea* strains at nitrite concentrations between 3 and 40 and even 70 mM, respectively, matches the extremely high half-saturation constant (K_m_) for nitrite determined for *Nl. hollandica* (Sorokin et al., 2012). The K_m_ of this strain was found to be 1 mM nitrite, which corresponds to the lowest substrate affinity of any NOB determined so far and is in line with the high substrate concentrations present in the bioreactor *Nl. hollandica* was isolated from. The *Nitrolancea* cultures investigated here revealed a higher affinity for nitrite, although there was a great variance between values for strain Z and only one measurement was done for *Nl. copahuensis*. Nevertheless, our preliminary results suggest that the K_m_ value of *Nitrolancea* is in the range of the different *Nitrobacter* species (Nowka et al., 2015). This higher substrate affinity fits with the presence of *Nitrolancea* in the geothermal area of Las Máquinas, where nitrite is rarely detected as usual for most natural systems. Nevertheless, high substrate concentrations are necessary for optimal growth of *Nitrolancea* and nitrite was oxidized slowly when present in low concentrations (Figures 4B,D).

Comparing the genomes and NxrA gene sequences from the different enrichments, it has become obvious that distinct representatives of *Nitrolancea* grew depending on the respective medium (for instance 3 mM vs. 60 mM nitrite for strains Z2 and Z4). Similarly, the representatives of *Nitrolancea* enriched from the Las Máquinas samples differed in the initial enrichments E2 and A4.4 in AOB and NOB medium, respectively (Supplementary Figure S4). The *nxrA* gene within the metagenome 40 of the culture E2 is non-operonal, whereas in close vicinity to the *nxrA* gene of the metagenome 35 a cytochrome *c* was found. Therefore, the initial samples must have contained a microdiversity of *Nitrolancea* strains, which selectively proliferate once their special demands were met.

*Nitrolancea*-like 16S rRNA gene sequences have only occasionally been detected in full-scale nitrifying activated sludge systems so far (Speirs et al., 2019). The *Nitrolancea*-typical marker fatty acid 12-methyl octadecanoic acid (18:0 12methyl) (Sorokin et al., 2012) was measured in low amounts (0.2% of total fatty acids) in activated sludge of the Hamburg-Dradenau WWTP by Kruse et al. (2013). The abundance of 12-methyl octadecanoic acid increased slightly to 0.7% after incubations with nitrite at 28°C and 32°C, which confirms the preference of *Nitrolancea* for elevated temperatures. However, similar glycolipids are also characteristic for other thermophilic *Chloroflexi*, like for instance *Thermomicrobium*, which has also been detected in WWTPs based on 16S rRNA gene sequences (Spieck et al., 2020). Therefore, it is still speculative if *Nitrolancea* also occurs in activated sludge, which was used as inoculum for the centrate reactor.

The geothermal area in Las Máquinas apparently offers beneficial conditions for *Nitrolancea*-like NOB, as revealed by our 16S rRNA amplicon data (Supplementary Figure S2, Supplementary Table S2) although closely related sequences have not been detected in this area previously (Urbieta et al., 2015a). Elevated ammonium or nitrite concentrations could not be measured at the sampling sites, thus posing the question if alternative substrates might have enabled proliferation of *Nitrolancea*. Some NOB are metabolically versatile and can use simple organic carbon compounds like formate, hydrogen, and even ammonia as additional energy sources (Koch et al., 2014, 2015; Daims et al., 2015; van Kessel et al., 2015). Furthermore, different NOB possess the genetic potential to use various sulfur compounds (Starkenburg et al., 2008; Lücker et al., 2013; Boddicker and Mosier, 2018; Kitzinger et al., 2018; Palomo et al., 2018) and the oxidation of sulfide has been demonstrated for *Nitrococcus mobilis* (Füssel et al., 2017). In the Las Máquinas area, a highly active sulfur cycle is present (Giaveno et al., 2013; Urbieta et al., 2014) and the ability of *Nitrolancea* species to oxidize thiosulfate should be tested in further studies. The ability for cyanate degradation might provide an alternative source to generate ammonia for N-assimilation.

In conclusion, we physiologically and genomically characterized three novel representatives affiliated with the genus *Nitrolancea*, which we enriched from a centrate treatment reactor at the WWTP Hamburg-Dradenau and the geothermal area Las Máquinas in Neuquen-Argentina. One of these strains represents a new species within the genus *Nitrolancea*. We provisionally refer to this strain as, *Candidatus* Nitrolancea copahuensis‘ (co.pa.hu.en’sis. N.L.fem.adj. copahuensis pertaining to the Copahue region in North-Patagonia, Argentina).

The strain is a mesophilic, aerobic, chemolithoautotrophic bacterium, which stoichiometrically converts nitrite to nitrate and uses carbon dioxide as carbon source. Forms short rods with tapered ends; Gram-positive. Requires ammonium supplementation and tolerates high nitrite and ammonium concentrations. Optimum growth temperature is 35–37°C. DNA G+C content is 62.7 mol%. Originated from a hot spring in the geothermal field Las Máquinas, Neuquen-Argentina.

## Data Availability Statement

The datasets generated for this study are available in the European Nucleotide Archive (ENA) under accession numbers ERS4399870, ERS4399871, ERS4399872, ERS4647270-74, ERS4648393-96, and LR794103-5. Strain availability will be performed upon request.

## Author Contributions

AG performed the sampling. ES, SK, and SL designed the research. SH worked on the *Nitrolancea* enrichments. ES and KS isolated the nitrite oxidizers and performed FISH. SK did physiological experiments. DI performed genome sequencing. MS, LK, and SL analyzed the data. ES, KS, SK, LK, AG, and SL wrote the manuscript. All authors read and agreed on the final manuscript.

## Conflict of Interest

The authors declare that the research was conducted in the absence of any commercial or financial relationships that could be construed as a potential conflict of interest.

## Footnotes

1 www.ncbi.nlm.nih.gov

2 http://rdp.cme.msu.edu
