# Supplementary material for: Defining Culture Conditions for the Hidden Nitrite-Oxidizing Bacterium Nitrolancea
Source: Front Microbiol. 2020 Jul 10;11:1522. doi: 10.3389/fmicb.2020.01522 (PMC7365893; doi:10.3389/fmicb.2020.01522)
Supplement: Supplementary file 1 [file Data_Sheet_1.PDF]

Supplementary Material

Supplemental Results and Discussion

Selective enrichment of Nitrolancea

No nitrite oxidation was observed by the initial centrate cultures at 46°C, but when incubated at 42°C with both ammonium and nitrite present, culture Z2.1 consumed 3 mM of nitrite within 4 months. A *Nitrolancea*-like 16S rRNA gene sequence could be detected in this culture by amplicon sequencing, but only constituted 1.4% of the reads (Table S2). After this culture was transferred to fresh medium, nitrite oxidation stagnated and the incubation temperature was lowered to 37°C (Z2.2). The relative abundance of *Nitrolancea*-like bacteria in this culture reached 23.5% after five months of incubation. Subsequent culturing at 60 mM nitrite (Z2.3.2) did not further increase the relative abundance of *Nitrolancea*, which reached 22.3% according to amplicon sequencing. Contrastingly, FISH demonstrated that cultivation at even higher substrate concentrations of 70 mM nitrite in culture Z2.3.3 resulted in almost exclusively lancet-shaped cells typical of *Nl. hollandica* (Figures 1a and 2a).

Nitrolancea abundances in the first Argentinian enrichment (A4.1) amounted to 14.1%. The subsequent culture A4.2 was transferred to 37°C, the nitrite concentration was elevated to 3 mM, and 3 mM ammonium was added. This resulted in an increase of *Nitrolancea*-like bacteria to 24.3% relative abundance (Table S2). While the abundance of this NOB did not change considerably upon a subsequent transfer under identical cultivation conditions (culture A4.3), a further increase of substrate (20 mM) combined with a high ammonium concentration (10 mM) finally led to a relative abundance of 43.6% of *Nitrolancea*-like bacteria in culture A4.4. Consequently, nitrite was increased to 30 mM in culture A4.5.1, which was also supplemented with 0.5 mM bicarbonate and 10 mM ammonium. Finally, the nitrite concentration was raised to 40 mM (A4.5.2), and a dilution series of this culture allowed obtaining an isolate (A.4.5.3).

The most abundant (46% based on 16S rRNA amplicon sequences) concomitant bacteria in the Argentinian hot spring-derived culture A4.4 were identified as members of the genus *Meiothermus*, which frequently have been reported to grow by nitrate reduction (Raposo et al., 2019). Cultures derived from the centrate treatment reactor (Z2.2 and Z2.3.2) also contained a high percentage of *Meiothermus* and additionally bacteria affiliated with the genus *Gemmatimonas*. Some *Gemmatimonas* species possess the ability to reduce nitrite to nitric oxide (Decleyre et al., 2016) and previous studies have shown that their relative abundance in Antarctic soil increased under N addition (Aanderud et al., 2018), even in oxic incubations. Likewise, in our enrichments their highest abundance was observed in culture Z2.3.2, which had a highly imbalanced ratio of nitrite to ammonium (60:1), although it remains to be determined how these bacteria would benefit from nitrate or nitrite reduction under oxic incubation conditions.

The highest enrichments of *Nitrolancea*-like bacteria from Argentina were achieved in media with a NO_2^-:NH_4^+ ratio of 1 to 2, as reflected by the high relative abundance based on 16S rRNA gene sequencing of *Nitrolancea* in culture A4.4 (Table S2). From the centrate treatment reactor, *Nitrolancea* was selectively enriched to almost purity at the most extreme NO_2^-:NH_4^+ ratio of 70:1 according to FISH analysis (culture Z2.3.3, Figure 1). Here, the high nitrite concentration of 70 mM might have become toxic to the accompanying microflora (Philips et al., 2002). Similarly, *Nitrobacter* has been reported to tolerate nitrite concentrations of 60 mM (Sayavedra-Soto et al., 2015) and this trait thus appears to be facilitated by the cytoplasmic NXR system.

Surprisingly, *Nitrolancea*-like NOB were highly abundant in the enrichment culture E2, which was supplemented with ammonium as the only substrate. However, no known ammonia-oxidizing microbe could be detected by *amoA*-specific PCRs, 16S rRNA gene amplicon sequencing or metagenomics, and it thus remains unclear how the NOB were supplied with nitrite. A similar observation has been made recently for thermophilic nitrite-oxidizing *Choroflexi* enriched from hot springs in Yellowstone National Park (Spieck et al., 2020). These enrichments were also active in producing nitrate when only supplied with ammonium, but no known ammonia-oxidizing organism could be detected in the metagenomes. It has been speculated that other enzymes might be involved in the possibly unspecific oxidation of ammonia (Bédard and Knowles, 1989), like for instance the carbon monoxide dehydrogenase. All analyzed *Nitrolancea* genomes possess a CO dehydrogenase, but no ability to oxidize ammonia was observed for cultures of *Nitrolancea* so far.

References:

Aanderud, Z. T., Saurey, S., Ball, B. A., Wall, D. H., Barrett, J. E., Muscarella, M. E., et al. (2018). Stoichiometric shifts in soil C:N:P promote bacterial taxa dominance, maintain biodiversity, and deconstruct community assemblages. Front. Microbiol. 9, 1401. doi:10.3389/fmicb.2018.01401.

Bédard, C., and Knowles, R. (1989). Physiology, biochemistry and specific inhibitors of CH_4, NH_4^+, and CO oxidation by methanotrophs and nitrifiers. Microbiol. Rev. 55, 68-84.

Decleyre H., Kim H., Tytgat B., Willems A. (2016). Highly diverse nirK genes comprise two major clades that harbour ammonium-producing denitrifiers. BMC Genomics 17:155. doi:10.1186/s12864-016-2465-0.

Philips, S., Laanbroek, H. J., and Verstraete, W. (2002). Origin, causes and effects of increased nitrite concentrations in aquatic environments. Rev. Environ. Sci. Biotechnol. 1, 115-141. doi:10.1023/A:1020892826575.

Raposo, P., Viver, T., Albuquerque, L., Froufe, H., Barroso, C., Egas, C., et al. (2019). Transfer of *Meiothermus chliarophilus* (Tenreiro et al.1995) Nobre et al. 1996, *Meiothermus roseus* Ming et al. 2016, *Meiothermus terrae* Yu et al. 2014 and *Meiothermus timidus* Pires et al. 2005, to *Calidithermus* gen. nov., as *Calidithermus chliarophilus* comb. nov., *Calidithermus roseus* comb. nov., *Calidithermus terrae* comb. nov. and *Calidithermus timidus* comb. nov., respectively, and emended description of the genus *Meiothermus*. Int. J. Syst. Evol. Microbiol. 69, 1060-1069. doi:10.1099/ijsem.0.003270.

Sayavedra-Soto, L., Ferrell, R., Dobie, M., Mellbye, B., Chaplen, F., Buchanan, A., et al. (2015). *Nitrobacter winogradskyi* transcriptomic response to low and high ammonium concentrations. FEMS Microbiol. Lett. 362, 1-7. doi:10.1093/femsle/fnu040.

Spieck, E., Spohn, M., Wendt, K., Bock, E., Shively, J., Frank, J., et al. (2020). Extremophilic nitrite-oxidizing *Chloroflexi* from Yellowstone hot springs. ISME J. 14, 364-379. doi:10.1038/s41396-019-0530-9.

Supplementary Figures and Tables

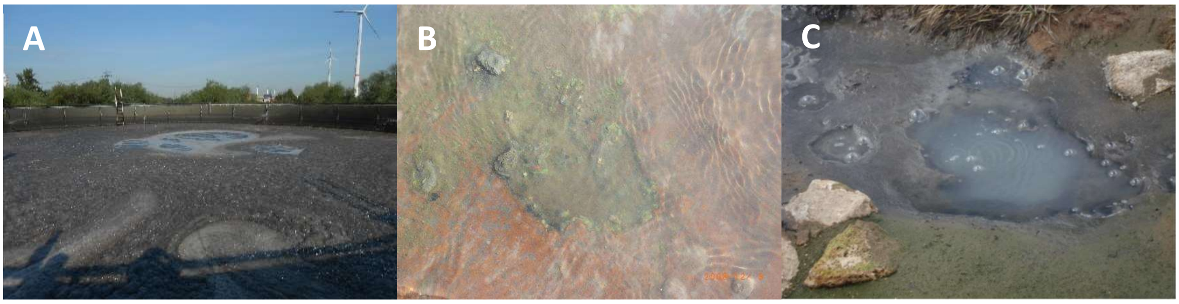

Figure S1: Sampling sites for (A) the centrate reactor Z2 and mud pools (B) E2 and (C) A4 in Las Máquinas, Neuquen-Argentina.

Table S1: Chemical composition of the samples from Las Máquinas.

Sample	Ca (ppm)	Cd (ppm)	Zn (ppm)	Fe (ppm)	Cu (ppm)	Na (ppm)	Ni (ppm)	Pb (ppm)	Co (ppm)	Mg (ppm)	Mn (ppm)	K (ppm)	Cr (ppm)	SO_4^{2-} (ppm)	Cl^- (ppm)	Conductivity (µS/cm)
**E2**	2.2	ND	ND	2.1	ND	2.2	ND	ND	ND	1.1	ND	0.8	ND	323.1	7.8	3210
**A4**	1.3	ND	ND	3.2	ND	1.7	ND	ND	ND	0.6	ND	2.2	ND	219	2.5	462

Table S2: Overview of the enrichment process for *Nitrolancea* derived from Las Máquinas (E2 and A4) and the WWTP in Dradenau-Hamburg (Z2). Cultures were inoculated with that of the previous date. The relative abundance of *Nitrolancea* and *Nitrospira* was obtained by 16S rRNA gene amplicon sequencing, a high increase is printed in bold.

Culture	Group	Incubation temperature	Inoculation date	Relative abundance %		Medium	Culture vessel	Metagenome
				Nitrolancea	*Nitrospira*			
E2	AOB	42°C	03.02.2011	**19.8**	0	0.5 mM NH_4^+	100 ml flask	35, 40
A4.1	NOB	42°C	23.03.2011	**14.1**	0	0.3 mM NO_2^-	250 ml bottle	51
A4.2	NOB	37°C	20.03.2018	**24.3**	0.3	3 mM NO_2^-/ 3 mM NH_4^+	300 ml flask	
A4.3	NOB	37°C	08.05.2018	23.2	0.3	3 mM NO_2^- / 3 mM NH_4^+	300 ml flask	
A4.4	NOB	37°C	20.06.2018	**43.6**	0.3	20 mM NO_2^- / 10 mM NH_4^+	300 ml flask	A2
A4.5.1	NOB	37°C	18.07.2018	-	-	30 mM NO_2^- / 10 mM NH_4^+/ 0.5 mM $NaHCO_3$	300 ml flask	
A4.5.2	NOB	37°C	18.07.2018	-	-	40 mM NO_2^- / 10 mM NH_4^+/ 0.5 mM $NaHCO_3$	300 ml flask	
A4.5.3	NOB	37°C	10^{-7} dilution of A4.5.2	-	-	0.5 mM NO_2^- / 0.5 mM NH_4^+/ 0.5 mM $NaHCO_3$	5 ml tubes	
Z2	Original sample	39°C	22.05.2017	0	0	-	-	
Z2.1	NOB	42°C	22.06.2017	1.4	0	3 mM NO_2 / 3 mM NH_4^+	300 ml flask	
Z2.2	NOB	42°C, 37°C (since 21.01.18)	23.10.2017	**23.5**	0	3 mM NO_2 / 3 mM NH_4^+	300 ml flask	Z2
Z2.3.1	NOB	37°C	29.03.2018	-	-	50 mM NO_2 / 1 mM NH_4^+/ 0.5 mM $NaHCO_3$	300 ml flask	
Z2.3.2	NOB	37°C	29.03.2018	22.3	0.1	60 mM NO_2 / 1 mM NH_4^+/ 0.5 mM $NaHCO_3$	300 ml flask	Z4

Z2.3.3	NOB	37°C	29.03.18	-	-	70 mM NO_2 / 1 mM NH_4^+/ 0.5 mM $NaHCO_3$	300 ml flask	
Z2.3.4	NOB	37°C	10^{-8} dilution of Z2.3.3	-	-	0.5 mM NO_2^- / 0.5 mM NH_4^+/ 0.5 mM $NaHCO_3$	5 ml tubes	
Z2.3.5	NOB	37°C	10^{-6} dilution of Z2.3.4			0.5 mM NO_2^- / 0.5 mM NH_4^+/ 0.5 mM $NaHCO_3$	5 ml tubes	

Table S3: FISH probes and PCR primers used in this study

Name	Sequence 5'-3'	Position[a]	FA/T_M[b]	Target group	Reference
16S rRNA-targeted FISH probes			FA [%]		
EUB338	GCTGCCTCCCGTAGGAGT	338-355	20	Most Bacteria	Amann et al., 1990
NON338	ACTCCTACGGGAGGCAGC	338-355	20	Negative control, complementary to EUB338	Wallner et al., 1993
Ntlc804	CAGCGTTTACTGCTCGGA	804-821	20	*Nitrolancea*	Sorokin et al., 2012
c1Ntlc804	CAGCGTTTACTGCGCGGA	804-821	20	-	Sorokin et al., 2012
c2Ntlc804	CATCGTTTACTGCTCGGA	804-821	20	-	Sorokin et al., 2012
c3Ntlc804	CAGCGTTTACTGCTAGGA	804-821	20	-	Sorokin et al., 2012
16S rRNA gene-targeted primers			T_M [°C]		
27F	AGRGTTYGATYMTGGCTCAG	8-27	50/56	All bacteria	Lane 1991
1492R	TACGGYTACCTTGTTACGACTT	1492-1513		All bacteria	Lane 1991
188f	CAAGGCCGATCAAGCAAA	188-197	55	*Nitrolancea*	Sorokin et al., 2012
1136r	TCTGGCTAGACATCCTCG	1136-1157	55	*Nitrolancea*	Sorokin et al., 2012
nxrA-targeted primers			T_M [°C]		
0031f	AAGGCACGCCAGTGGGAA GAGTTCTA	31-56	60	*Nitrolancea*	Sorokin et al., 2012
3598r	TTTCGCACYGCCACGTGCGTG TCCCG	3598-3623	60	*Nitrolancea*	Sorokin et al., 2012
nxrB-targeted primers			T_M [°C]		
NxrB169f	TACATGTGGTGGAACA	169–184	56	All *Nitrospira* lineages	Pester et al., 2014
NxrB638r	CGGTTCTGGTCRATCA	638–653	56	All *Nitrospira* lineages	Pester et al., 2014

[a]Probe binding position according to the *E. coli* 16S rRNA, *Nitrobacter hamburgensis* X14 *nxrA* (Nham_0951), *Nitrospira moscoviensis nxrA* (NITMOv2_0255), *Nitrosomonas europaea amoA* (NE0944), or *Nitrososphaera viennensis amoA* (NVIE_027270) gene numbering.

[b]FA, Formamide concentration used in hybridization buffer; T_M, temperature used for primer annealing.

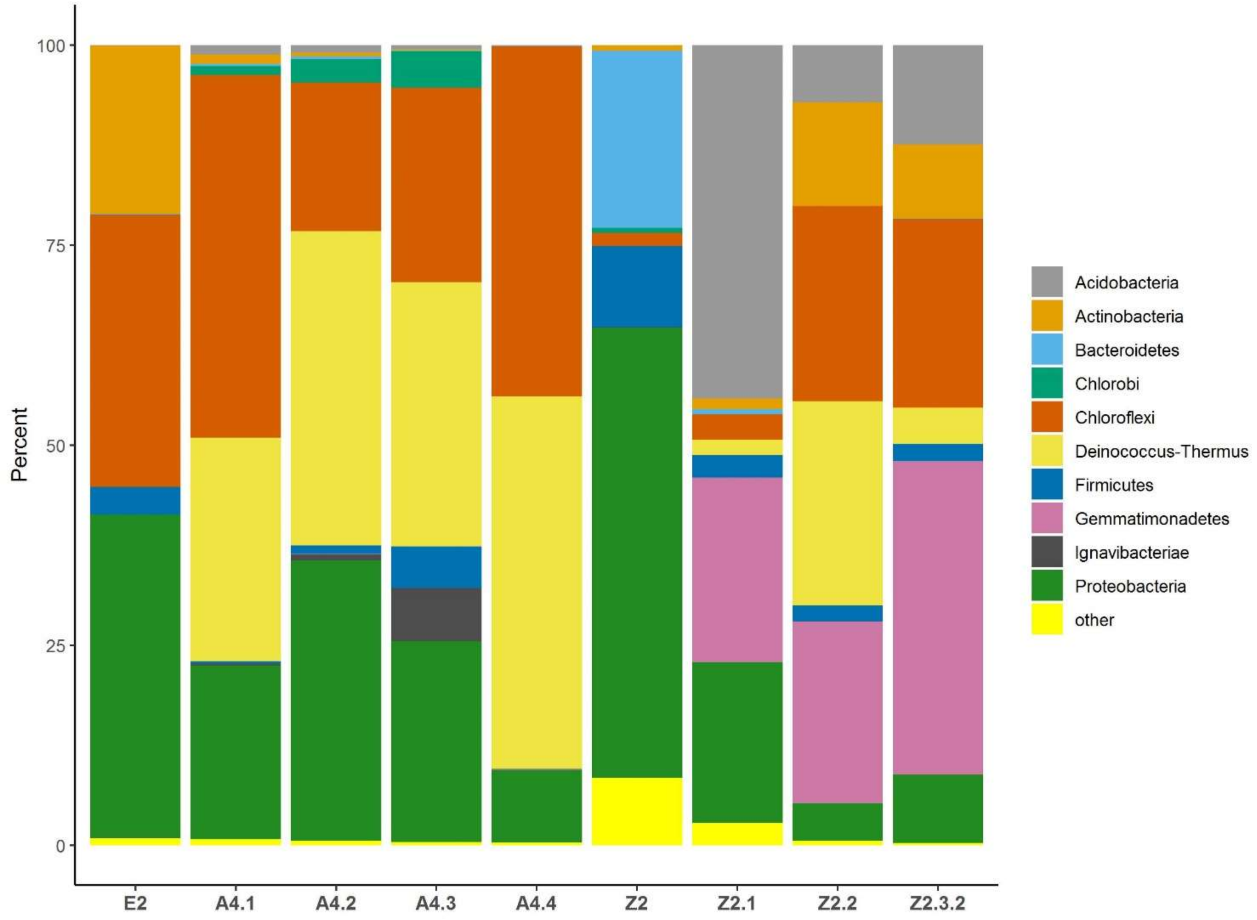

Figure S2: 16S rRNA gene amplicon sequencing results. Relative abundance of bacterial phylum-level OTUs present in ammonia-oxidizing (E2) and nitrite-oxidizing cultures (A4.1 - A4.4) enriched from Las Máquinas or a centrate treatment reactor (Z2.1 - Z2.3.2). DNA from sample Z2 was isolated directly after sampling.

Table S4: Growth parameters and kinetic constants of *Ca.* Nitrolancea copahuensis (A5.4.2) and *Nl. hollandica* strain Z* (Z2.3.4).

Parameter	A4.5.2[1]			Z2.3.4[2]	
	a	b	c	a	b
Generation time (G) in days (calculated with cells)	1.8	4.9	2.2	13.8	3.6
Generation time (G) in days (calculated with protein)	2.5	3.6	2.4	2.8	2.2
Maximum specific cell activity (V_{max_cell}) in fmol NO_2 cell^{-1} h^{-1}			1.25	1.18	0.57
Growth yield (Y_p) in mg protein mmol nitrite^{-1}	0.88	0.25	0.39	0.17	0.21
Growth yield (Y_c) in LOG[cells] mmol nitrite^{-1}	9.40	9.40	9.53	8.82	9.40
Maximum specific activity (V_{max}) in µmol nitrite mg protein^{-1} h^{-1}			26.76	14.12	11.12
Saturation constant for activity (K_m) in µM nitrite			215	453	116

*Without differentiation between the genotypes Z2 and Z4

[1]a to c, biological triplicates

[2]a and b, biological duplicates

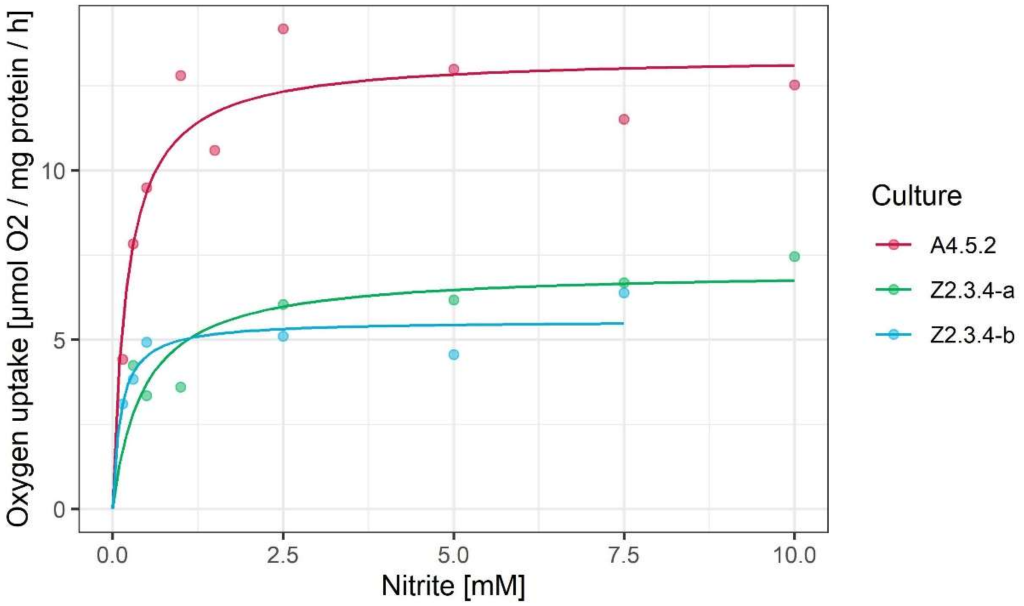

Figure S3: Nitrite oxidation kinetics of *Ca.* Nitrolancea copahuensis (culture A4.5.2) and *Nl. hollandica* strain Z (culture Z2.3.4). Michaelis-Menten plots of oxygen uptake at different nitrite concentrations are shown. The kinetic parameters were calculated by fitting a Michaelis-Menten kinetic to the data. Values for V_{max} and K_m are shown in table S4.

Table S5: 16S rRNA (blue) and *nxrA* (green) gene identities (in %) of the three new *Nitrolancea* strains.

Genome	A2	Z2	Z4	*Nl. hollandica*
**A2**	100	98.57	98.44*	98.5
**Z2**	92.12*	100	100*	99.93
**Z4**	94.41	94.63*	100	100*
Nl. hollandica	97.17	94.07*	98	100

*only partial sequence available for 16S rRNA of Z4 (898 bp) and *nxrA* of Z2 (2028 bp).

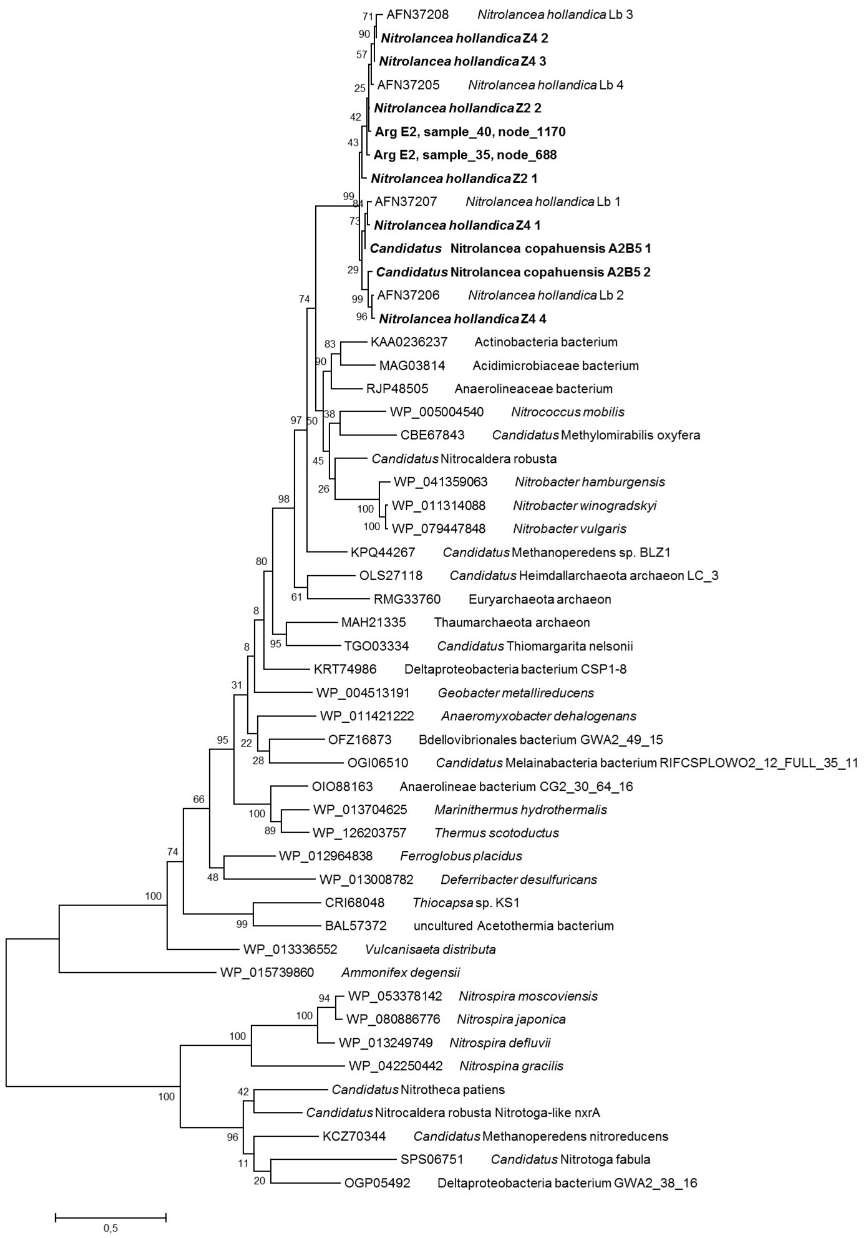

Figure S4: NxrA-based phylogenetic affiliation of the *Nitrolancea* strains identified in the centrate treatment reactor and Argentinian enrichment cultures. The maximum likelihood tree is based on an alignment containing 1605 valid amino acid positions. Statistical branching support values based on 1000 bootstraps are indicated on the nodes. Sequences obtained in this study are depicted in bold. The scale bar represents the expected changes per amino acid position. For designation of cultures see Table S2. The different NxrA copies are numbered 1 – 4, with 1 used for the operonal NxrA.

Table S6: Average nucleotide identities of the four *Nitrolancea* strains.

Genome	A2	Z4	Z2	*N. hollandica*
**A2**	100	92.25	92.28	91.93
**Z4**	92.25	100	99.97	99.19
**Z2**	92.28	99.97	100	99.25
Nl. hollandica	91.93	99.19	99.25	100

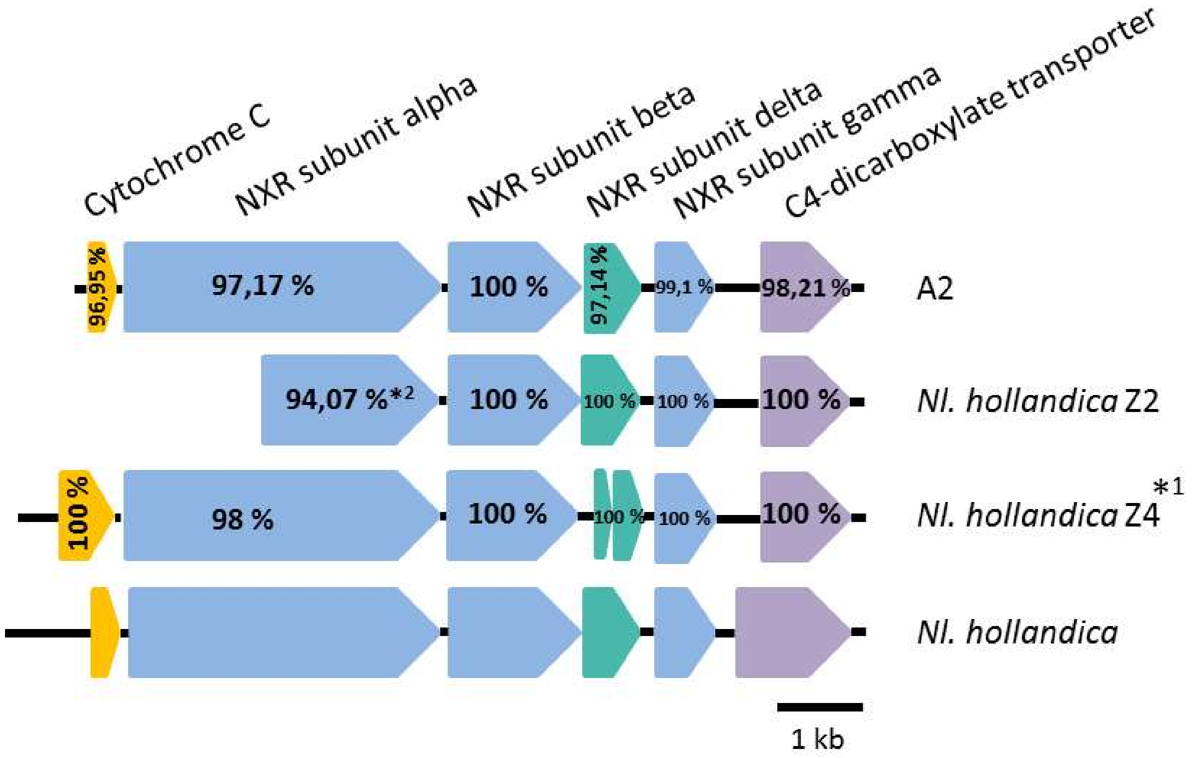

Figure S5: Nitrite oxidoreductase operon structure. Genomic organization of the nitrite oxidoreductase (NXR) operon revealing protein identities of the NXR subunits in the recovered *Nitrolancea* genomes in comparison to the reference strain *Nl. hollandica*. *[1] inverted orientation *[2]only partial sequence available.
